# Co-digestion approach for enhancement of biogas production by mixture of untreated napier grass and industrial hydrolyzed food waste

**DOI:** 10.3389/fbioe.2023.1269727

**Published:** 2024-01-08

**Authors:** Jayen Aris Kriswantoro, Kuan-Yin Pan, Chen-Yeon Chu

**Affiliations:** ^1^ Ph.D. Program of Mechanical and Aeronautical Engineering, Feng Chia University, Taichung, Taiwan; ^2^ Institute of Green Products, Feng Chia University, Taichung, Taiwan; ^3^ School of Life Sciences and Technology, Bandung Institute of Technology, Bandung, Indonesia; ^4^ Department of Materials Science and Engineering, College of Engineering and Science, Feng Chia University, Taichung, Taiwan; ^5^ National Research Council of Italy, Institute of Atmospheric Pollution Research, Rome, Italy

**Keywords:** anaerobic digestion, biogas, co-digestion, food waste, napier grass

## Abstract

The co-digestion of untreated Napier grass (NG) and industrial hydrolyzed food waste (FW) was carried out in the batch reactor to investigate the effect of substrate ratios on biogas production performance. Two-stage anaerobic digestion was performed with an initial substrate concentration of 5 g VS_added_/L and a Food to Microorganism Ratio (F/M) of 0.84. The 1:1 ratio of the NG and FW showed the optimum performances on biogas production yield with a value of 1,161.33 mL/g VS_added_ after 60 days of digestion. This was followed by the data on methane yield and concentration were 614.37 mL/g VS_added_ and 67.29%, respectively. The results were similar to the simulation results using a modified Gompertz model, which had a higher potential methane production and maximum production rate, as well as a shorter lag phase and a coefficient of determination of 0.9945. These findings indicated that the co-digestion of Napier grass and hydrolyzed food waste can enhance biogas production in two-stage anaerobic digestion.

## 1 Introduction

Environmental pollution, including the emission of carbon dioxide (CO_2_), and shortage of energy supply have affected human health and life, stimulating research on renewable energy for a better life. Nowadays, there is an abundance of reports showing that the anaerobic digestion reaction system for biogas production is a feasible and cost-effective way for energy recovery from various resources including lignocellulosic material such as food waste and any agricultural biomass waste (Azam et al., 2023). Anaerobic digestion (AD) is a biological process that breaks down organic substrates and converts them to methane (CH_4_) and carbon dioxide (Wu Q. et al., 2022). Methane is a high heating value (55.5 MJ/kg) energy resource, and the relative abundance in biogas is about 55%–75%. Biogas is considered to become an important sustainable renewable energy sources in the future (Arutyunov et al., 2022). However, an in-depth understanding of efficient renewable energy production through a biological process in combination with low-cost implementation is absolutely necessary. Therefore, biogas production from organic wastes has been reported in many studies (He C. et al., 2022; Al Ajlouni, 2022; Norouzi and Dutta, 2022; Simioni et al., 2022). According to previous reports, the cellulose and hemicellulose contained in AD feedstocks must be hydrolyzed to small sugars such as glucose, xylose, mannose, and galactose. These sugars can then be used as carbon sources for methanogenesis in the final stage of anaerobic digestion on biomethane production (Gomes et al., 2022; Soltaninejad et al., 2022; Thongbunrod and Chaiprasert, 2023; Yadav et al., 2023).

As the most abundant organic waste on earth, food waste includes household, food processing, canteen, and restaurant waste, which mainly contains carbohydrates such as starch and sugar, proteins and lipids. With this composition, food waste had great potential for biogas production using the AD system and the benefits of organic waste reduction (Cohn et al., 2022; Onyeaka et al., 2022; Qian et al., 2022). On the other hand, Napier grass, which is classified as lignocellulosic biomass, has cellulose, hemicellulose, and lignin as its main organic components. This lignocellulosic biomass can be used as a feedstock for biogas production, potentially combined with food waste in the AD system (Donkor et al., 2022; Govarthanan et al., 2022; Yadav et al., 2023). Napier grass, a perennial C-4 grass species indigenous to Africa, has been cultivated in numerous tropical regions, including various countries in Southeast Asia for ruminant feed. Currently, research focused on generating bioenergy and environmentally friendly bio-based products has intensified, particularly exploring second-generation feedstock that comprises perennial grasses (Thaemngoen et al., 2020; Rakau et al., 2022). Switchgrass and *Miscanthus* sp. have attracted researchers in America and Europe, respectively, while Napier grass has become more appealing to many Asian countries due to its benefits (Pomdaeng et al., 2022). Napier grass is classified as biomass with high lignocellulosic content (>34%) (Manokhoon and Rangseesuriyachai, 2020; Gundupalli et al., 2023), resulting in high production yield (40–87 tons per 10,000 m^2^/year) and low overall production cost (Thaemngoen et al., 2020; Pomdaeng et al., 2022; Balakrishnan et al., 2023). It is advantageous because of its ease of harvesting and fast growth, requiring low attention for plantation and nutrient demand (Gundupalli et al., 2023). The organic nutrient from the feedstock is converted into biogas biologically through the following four steps: hydrolysis, acidogenesis, acetogenesis, and methanogenesis (Akcakaya et al., 2022). Mono-anaerobic digestion may be less efficient for biogas production, and mixing the two materials into a co-digestion system can help increase biogas production. The use of a single substrate in mono-digestion, in some cases, showed lower methane production yield due to the lack of nutrients to support the methanogenesis process by methanogen (Choudhary et al., 2022; Jiang et al., 2022).

Several co-digestion systems have been studied, including waste paper slurry, sewage sludge, algae, and rice straw (Liu H. et al., 2022; Kaushal et al., 2022; Xu et al., 2022; Sawasdee et al., 2023). Various analyses have been performed on these substrates, such as the effect of C/N (Carbon-to-Nitrogen) ratio measurements on biogas production, with high C/N ratios leading to increased nitrogen use efficiency (NUE) in cellulosic materials. This condition could increase the biomass produced in the digester while extending the cellulose digestion times (Yong et al., 2015; Huang et al., 2022). The operating temperature affects the performance of the reactor by providing a suitable condition for methanogen growth. Most methanogens are mesophiles, they overgrow at mesophilic temperatures and convert a higher proportion of organic matter in the mesophilic temperature range (Rahman et al., 2021). Therefore, the co-digestion of untreated Napier grass and hydrolyzed food waste was examined in mesophilic conditions in this study. Food waste hydrolysate (FW) is a complex organic material that possesses inhibitory compounds for anaerobic digestion, including phenolic compounds and other preservative residues found in food (Pomdaeng et al., 2023). Due to its protein and lipid richness, the FW encounters methane generation challenges as it has the potential to inhibit methanogenesis activities (Xu et al., 2020). The protein-rich feedstock leads to the accumulation of ammonia that can interfere with the methanogen’s enzyme activity also causing the proton imbalance and potassium deficiency inside the bacterial cell (Milán et al., 2010; Xiao et al., 2022). The lipid content in AD, especially the long-chain fatty acids (LCFAs), acts as a limiting factor of lipid degradation for methane production (He X. et al., 2022). The co-digestion of food waste with untreated Napier grass has the potential to address the aforementioned challenges.

Currently, there are many studies on the co-digestion of food waste with other feedstocks, including various sludge and animal manure (Liu H. et al., 2022; Abbas et al., 2023). Few reports have been published on biogas production by co-digestion of food waste (FW) with Napier grass (NG). However, Napier grass and food waste are abundant in many areas, especially in tropical regions, that can be easily harvested and obtained for further processing. Aside from the benefits, Napier grass contains over 34% cellulose content, compared to hemicellulose (18%–21.4%) and lignin (6%–23.4%) contents (Manokhoon and Rangseesuriyachai, 2020). Therefore, the potential of the feedstock in bioenergy generation via anaerobic digestion depends on its cellulose and hemicellulose contents. The profile of Napier grass holds significant potential to be studied for biogas generation. Most reported studies have focused on using treated Napier grass to produce biogas, including chemical pretreatment with sodium hydroxide or deep eutectic solvents (Pinpatthanapong et al., 2022; Gundupalli et al., 2023), biological pretreatment with mixed cultures enzyme (Rangseesuriyachai et al., 2023), and physical pretreatment with sonication (Song et al., 2023). However, there are limited studies on the use of untreated Napier grass. Only one study reported the performance of untreated Napier grass and food waste (without hydrolysis process) co-digestion in repeated batch fermentation that only targeted methane as the final product. The optimal C/N ratio of the feedstocks in single-stage repeated batch fermentation was suggested by yielding the methane with a value of 411 mL/g VS_added_ at a ratio of 1:4 (NG:FW) (Boonpiyo et al., 2018). Subcritical water hydrolysis (SCWH) was used in this study to pretreat food waste. The method’s unique features were implemented for optimal results in biogas production, thereby enhancing the novelty of this study. The use of two-stage anaerobic digestion becomes the focus of this study to explore the effect of different ratios of Napier grass and food waste on biohydrogen and methane production performance. As a novel way of enhancing the biogas generation performances, the two-stage digestion approach applied in this study performs the co-digestion of interesting feedstocks. The two-stage anaerobic digestion adopted from previous studies (Pomdaeng et al., 2022; Kriswantoro et al., 2023), that worked with bioplastic waste and Napier grass, had two separate processes. The first stage focused on the production of biohydrogen and organic acids using hydrogen-producing bacteria as inoculum, while the second stage’s primary purpose was methane production by methanogens in the mixtures. In addition to total solids (TS), volatile solids (VS), and soluble chemical oxygen demand (SCOD), this study investigated the change in volatile fatty acid (VFA) concentration after fermentation. The kinetic study using the modified Gompertz model was also performed to predict the effect of NG/FW ratio on biogas production in batch reactors.

## 2 Materials and methods

### 2.1 Substrates and inoculum

The substrate of this study was mixed with raw Napier grass and food waste hydrolysate. The raw Napier grass (*Pennisetum purpureum*) (NG) was collected from Nantou, Central Taiwan. The chopped fresh Napier grass was dried and ground into powdery size. Food waste (FW) mainly contained cooked food residues such as rice, noodles, meat, fish, and vegetables which were collected from various places in Taichung City. The food waste and water were mixed at a ratio of 1:1 and then transferred to the reactor for subcritical water (SCW) pretreatment in 70%–75% of the working volume. The SCW hydrolysis was carried out for 30 min by Riqian Development Co., Taiwan at a pressure of 1.9–3.5 bar and a temperature range of 119°C–195°C. This SCWH method was adapted from a previous study (Chen et al., 2023). The slurry of food waste hydrolysate was sealed in plastic bags and stored in a freezer at −20°C.

The mixed culture of hydrogen-producing bacteria was used as the inoculum in the first stage for biohydrogen production which was provided by the Institute of Green Product (IGP), Feng Chia University. The sludge from anaerobic digestion treating the swine manure was used as methane inoculum of the second stage in this study, which was collected from a pig farm, in Nantou, Central Taiwan. The characteristics of substrates and inoculum were shown in Table 1.

**TABLE 1:** Characteristic of substrate and inoculum.

Parameters	NG	FW	Hydrogen inoculum	Methane inoculum
Total Solids (TS) (%)	92.9 ± 0.2	9.0 ± 0.1	5.9 ± 0.04	10.8 ± 0.1
Volatile Solids (VS) (%)	84.4 ± 0.6	7.2 ± 0.1	5.5 ± 0.03	5.17 ± 0.2
VS/TS (%)	90.8 ± 0.01	79.6 ± 0.01	93.2 ± 0.01	47.9 ± 0.01
Total Carbohydrate (%)	68.9 ± 0.01	39.3 ± 0.22	NA	NA
C/N Ratio	65.8 ± 3.29	68.9 ± 0.12	NA	NA
Cellulose (%)	41.3 ± 1.9	NA	NA	NA
Hemicellulose (%)	14.3 ± 1.2	NA	NA	NA
Lignin (%)	13.2 ± 1.7	NA	NA	NA
Phenolic content (mg/L)	NA	11.3 ± 0.01	NA	NA
Grease content (%)	NA	0.58 ± 0.04	NA	NA

NA (Not Applied)

### 2.2 Anaerobic digestion in batch reactor

The anaerobic digestion was conducted in a two-stage batch using a vial bottle as a reactor with an initial F/M ratio (Food-to-Microbe ratio) and substrates concentration was set at 0.84 and 5 g VS_added_/L, respectively. In the co-digestion reactor, the mixture ratio (in gram of VS) between Napier grass (NG) and food waste (FW) labeled as A, B, C, D, E, NG, and FW which represent NG: FW ratio of 4:1, 3:2, 1:1, 2:3, 1:4, 5:0 and 0:5, respectively. The mixture of inoculum and distilled water were used as a control. The glass vial bottles were then sealed with rubber and metal sealers. The sealed bottles were flushed with Argon gas (inert gas) for 2 min to achieve anaerobic conditions. The first stage was carried out for about 2 days and then was changed to the second stage by swine manure inoculation (F/M ratio 0.84 in grams of VS) as the source of methanogens, and the pH was adjusted to 7. The characteristics of the different two-stage experiments are shown in Table 2. In this study, all experiments were performed in duplicate.

**TABLE 2:** Initial SCOD, pH and VS in different ratio of NG and FW with final condition after methane production.

Sample code	Hydrogen production step					Methane production step							
	NG:FW^a^	Initial SCOD^b^ (g/L)	Initial pH	Initial VS (g/L)	H_2_ yield^c^	Initial SCOD^b^ (g/L)	Final SCOD^b^ (g/L)	SCOD^b^ removal (%)	Initial pH	Initial VS (g/L)	Final VS (g/L)	VS removal (%)	CH_4_ yield^c^
A	4:1	5.7 ± 0.1	5.5 ± 0.03	5 ± 0.07	0.2 ± 0.05	4.6 ± 1.8	3.2 ± 1.6	30.5 ± 1.6	7.0 ± 0.01	16.31 ± 1.2	12.4 ± 0.5	24.0 ± 0.9	220 ± 12
B	3:2	6.1 ± 0.2	5.6 ± 0.03	5 ± 0.07	0.4 ± 0.01	5.9 ± 1.3	3.2 ± 0.8	46.4 ± 1.2	7.0 ± 0.01	16.44 ± 1.5	13.1 ± 0.5	20.3 ± 1.0	185 ± 13
C	1:1	6.6 ± 0.1	5.5 ± 0.03	5 ± 0.07	0.4 ± 0.03	5.6 ± 2.4	3.1 ± 1.0	45.1 ± 0.9	7.0 ± 0.03	14.42 ± 0.4	13.6 ± 0.5	5.7 ± 0.5	235 ± 19
D	2:3	7.0 ± 0.1	5.6 ± 0.03	5 ± 0.07	0.5 ± 0.04	6.8 ± 2.4	3.7 ± 1.5	45.0 ± 1.0	6.8 ± 0.01	16.35 ± 0.4	8.1 ± 1.5	50.8 ± 1.0	180 ± 18
E	1:4	6.8 ± 0.1	5.5 ± 0.03	5 ± 0.07	0.5 ± 0.01	6.3 ± 1.1	2.0 ± 1.2	68.5 ± 0.5	7.0 ± 0.01	14.33 ± 0.4	14.5 ± 0.4	1.6 ± 0.4	187 ± 2
NG	5:0	4.0 ± 0.1	5.5 ± 0.03	5 ± 0.04	0.2 ± 0.05	6.4 ± 1.5	3.4 ± 0.9	46.0 ± 1.0	7.3 ± 0.01	15.18 ± 1.5	11.3 ± 0.9	25.6 ± 1.2	183 ± 2
FW	0:5	8.8 ± 0.1	5.5 ± 0.03	5 ± 0.07	0.4 ± 0.04	8.1 ± 1.5	3.8 ± 2.4	50.4 ± 4.9	7.0 ± 0.01	15.27 ± 0.0	14.5 ± 1.0	0.5 ± 0.5	168 ± 11
Control	NA	3.8 ± 0.5	5.5 ± 0.03	1.2 ± 0.1	0.02 ± 0.0	8.6 ± 0.0	4.1 ± 0.8	48.7 ± 4.6	8.1 ± 0.01	1.4 ± 0.03	0.86 ± 0.2	38.6 ± 0.1	213 ± 18
Sucrose	40 g/L	17.0 ± 0.3	5.5 ± 0.03	2.3 ± 0.1	19.7 ± 0.9	NA	NA	NA	NA	NA	NA	NA	NA

^**a**^ Ratio of NG:FW, in g VS_added_.

^**b**^ Soluble Chemical Oxygen Demand; NA (Not Applied).

^**c**^ Production yield in mL/g COD.

### 2.3 Analytical methods

The SCOD, TS, TSS (Total Suspended Solid), VS, VSS (Volatile Suspended Solid), and pH were analyzed according to the standard method (American Public Health Association (APHA), 1998). In brief, SCOD was detected by using potassium dichromate reagent and sulfuric acid reagent. Before testing, the sample was centrifuged for 5 min and filtered using the 0.45 µm membrane filter. TS and VS were measured after the sample was dried at 105°C and 550 °C in the oven (Precision Oven Model JA-72) and furnace (Model KEO-27L), respectively. The pH was determined by a pH meter (Thermo Scientific Orion Star A111). For the biogas composition was measured by gas chromatography (GC) with a Thermal Conductivity Detector (TCD) (GC System HP 6890 Series, USA). The temperature of the injector and detector were set at 175 °C and 250 °C, respectively. Helium (He) was used as the carrier gas at a flow rate of 40 mL/min. High-Performance Liquid Chromatography (HPLC-CM5000 series, Japan) from HITACHI was used to examine the volatile fatty acids (VFAs) concentration in the digester mixture.

## 3 Results and discussions

### 3.1 Biogas production performance

**FIGURE 1:**
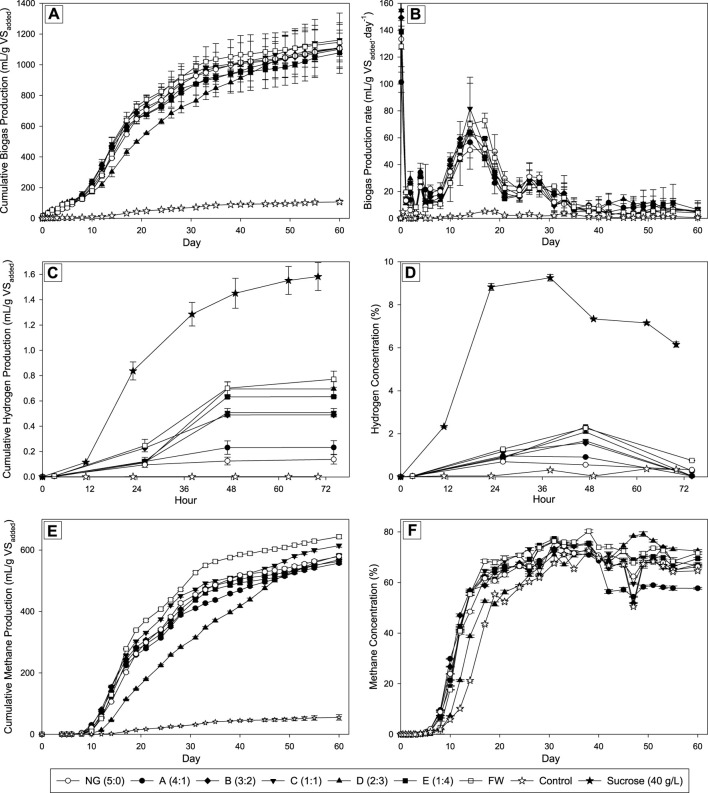
Biogas production performance with different ratios of NG and FW. **(A)** Cumulative Biogas Production (mL/g VS_added_); **(B)** Biogas Production Rate (mL/g VS_added_ per day); **(C)** Cumulative Hydrogen Production (mL/g VS_added_); **(D)** Hydrogen Concentration (%); **(E)** Cumulative Methane Production (mL/g VS_added_); **(F)** Methane Concentration (%).

The performances of the biogas production from the co-digestion of Napier grass (NG) and food waste (FW) at different mixing ratios were shown in Figure 1. The cumulative biogas production results (see Figure 1A) showed that the production increased significantly after 8 days of incubation. This trend was observed in all fermentation conditions except the control due to the lack of nutrients to support the bacterial growth in the reactor. The increase in cumulative biogas production remained significant up to 38 days of incubation. Supported by the biogas production rate data (see Figure 1B), the highest rate was reached after 14 days of incubation and then decreased dramatically until day 40 with a value of less than 10 mL/g VS_added_ per day. After day 40, the production rate decreases steadily until the end of fermentation after 60 days of incubation at 37°C. In the first 8 days of incubation, including the 2 days of the first stage and the early stage of the second stage, the increased value of the biogas produced was insignificant. The content of biohydrogen in the biogas produced in the first stage was also low. The results in Figures 1C, D showed the cumulative biohydrogen production after 2 days below 1 mL per gram VS_added_ with hydrogen concentration below 2.5%, which were much lower compared to the control (using sucrose as the main substrate). The sucrose in the control is less complex than any other sugar or compound found to be abundant in the NG and FW mixture, as it is a disaccharide compound. This led to the highest cumulative production and concentration of hydrogen from sucrose. The inoculum utilized in this study was collected from the mother tank reactor that had been fed with sucrose at a concentration of 40 g/L and 12 h of hydraulic retention time (HRT). Since the reactor had reached a steady state, the bacteria within it had sufficiently adapted to produce hydrogen using sucrose as the primary substrate. Additionally, the dominant strains in the initial stage’s inoculum were *Ethanoligenens* sp. (approximately 30%), *Bifidobacterium* sp. (approximately 23%), and *Sporolactobacillus* sp. (approximately 18%) (data not presented in this study). Those bacteria genera were reported in biohydrogen reactors that showed a good ability for hydrogen production together with sucrose consumption (Michelz Beitel et al., 2017; Li et al., 2019; Chu et al., 2021). The aforementioned conditions corroborate the findings of this study, which demonstrated that sucrose yielded the highest hydrogen production in comparison to the co-digestion of NG and FW. However, the involvement of lactic acid bacteria in the hydrogen production reactor is still debatable due to some findings mentioning their ability to inhibit the hydrogen production rate by competing the sugar consumption with other hydrogen-producing bacteria (Dzulkarnain et al., 2022).

In the first stage, the hydrolysis of the complex substance in the mixture including cellulose, hemicellulose, protein, and fats has susceptibly happened produced fermentable sugar and other less complex substances. Those components will be utilized by bacteria cells for their growth while producing organic acid that acidifies the mixtures also another important desired product, is biohydrogen (H_2_) (Li et al., 2010). Due to the untreated Napier grass used in this study, a complex substance remained in the mixtures making it difficult to produce hydrogen via digestion. The mono-digestion of NG exhibited the lowest hydrogen concentration with a value of 0.6%, while all co-digestions accounted for higher values ranging between 0.9% and 2.3%. Despite the possible improvement of hydrogen production via co-digestion with food waste hydrolysate, the production results remained low. Lower production of hydrogen in the FW may be attributed to the protein and fat compositions, as reported in previous studies (Yun et al., 2018; Sohrab Hossain et al., 2023). The previous study has reported the 7 days lag phase on co-digestion of *Sargassum* spp., categorized as lignocellulosic biomass, and organic domestic waste. This lag phase was observed due to the bacteria from the anaerobic sludge that treating the swine manure needing time to adapt to the new condition that was rich in organic acid and undigested materials in the first stage. This study has a similar lag phase period and in line with the previously reported study (López-Aguilar et al., 2023).

All conditions reached the peak hydrogen concentration after 48 h of incubation. The observed hydrogen concentration correlated with the amount of food waste in the mixtures. As the proportion of food waste hydrolysate in the mixture increased while decreasing for the untreated Napier grass, so did the hydrogen concentration in the produced biogas after 2 days of fermentation. Based on these results, Table 2 shows that only Napier Grass achieved the lowest H_2_ yield (mL/g COD) compared to only FW and all co-digestions. Among all the co-digestions, the NG:FW ratio of 4:1 resulted in the lowest H_2_ yield compared to other conditions that generated higher hydrogen yield with a higher FW ratio in the mixture. It made sense due to the higher fermentable sugar in the higher proportion of pretreated food waste with subcritical water compared to untreated Napier grass. The subcritical water pretreatment was reported to be a promising approach to hydrolyze the food waste before involving it as substrate in anaerobic digestion (Chen et al., 2023). The findings in this study could support the previous study for involving emerging technology in energy recovery from waste through anaerobic digestion. In this study, the C/N ratio was not found to be the main factor affecting hydrogen production performance. This is because both substrates investigated had similar C/N ratios (see Table 1). Previous studies have shown that C/N ratios significantly affect hydrogen biosynthesis. Different C/N ratios have been found to influence the metabolic pathway inside dark fermentation, which is related to hydrogen production performance (Litti et al., 2022).

The second stage of the anaerobic digestion system used in this study was mainly aimed at methane production from these substrate mixtures. The methanogens from the swine manure used as inoculum can potentially be divided into two main groups based on the methanogenesis pathway. The first, hydrogenotrophic methanogenesis, is the biosynthetic pathway that converts hydrogen (H_2_) and carbon dioxide to methane. The second pathway named acetoclastic methanogenesis which the methanogens could convert acetate into methane and carbon dioxide (Nikitina et al., 2022). A two-stage anaerobic digestion process may offer benefits for attaining optimal conditions for producing both hydrogen and methane. The first stage focuses on acidification, but methanogenesis is inhibited by acid accumulation in acidic environments. Higher acid production was found to be correlated with increased hydrogen generation in the first stage. The highest NG ratios (A; 4:1 of NG:FW) resulted in the lowest hydrogen production yield, as previously mentioned. However, the A conditions were able to produce significantly higher methane yield compared to all conditions except for the 1:1 ratio, with a value of 220 mL/g COD (see Table 2). A higher proportion of FW in the mixture provided more fermentable sugars for hydrogen production, but also resulted in the production of organic acids as a byproduct. However, excessive organic acid production in this stage is undesirable for the methanogenesis stage as it may inhibit the methanogens. The second stage reactor facilitates methanogen maturation in a neutral environment, separated from hydrogen and acid production (Chu et al., 2020; Saidi et al., 2023). The performance of methane production in various conditions of the NG: FW ratio was shown in Figures 1E, F. All the ratios examined in this study observed similarity in reaching the logarithmic phase of methane generation. After 10 days of incubation, the cumulative methane production significantly increased until day 40. The data on methane concentration also supported this observation that the methane proportion in the biogas produced sharply increased after 10 days of incubation and remained increased until reached the peak on day 30. The methane concentration was maintained at a constant level of 65%–80% until the end of the fermentation process. This finding was in line with the previous report that biogas produced from anaerobic digestion is composed of 55%–65% biomethane, 45% carbon dioxide, and 1%–2% other gases (Antukh et al., 2022).

The maximum methane yield in the co-digestion system, 614.37 mL/g VS_added_, at the optimal Napier grass to food waste ratio of 1:1, was 6% higher than that obtained from Napier grass alone in the mixtures. This finding means that among the co-digestion conditions (A to E), condition C (ratio of 1:1) had relatively the highest yield of methane production, which is the optimal ratio for co-digestion of these two substrates. According to these results, the untreated Napier grass was less susceptible to AD compared to the food waste hydrolysate due to the high content of complex substances, including cellulose (41%), hemicellulose (14%), and lignin (13%) (see Table 1). These findings were in line with the previous studies that the rate of the hydrolysis step in the AD process was influenced by the composition of the substrate used to produce methane (Boonpiyo et al., 2018). The 1:1 ratio yielded the highest amount of methane, with a value of 235 mL/g COD (refer to Table 2). This is 28.4% and 39.9% higher than the methane yield of the mono-digestions of NG and FW, respectively. This ratio helped to mitigate the negative effects of acidic accumulation in the first stage and created a more favorable environment for methanogens to produce methane. The second stage mixture contained the complex nutrients (mainly derived from NG), which were continuously degraded and supplied sufficient material for methane generation.

The methane production performances identified in this study were compared to previous reports, as shown in Table 3. Compared to other reports working on the co-digestion of NG and FW, this study produced the highest methane yield (mL/g VS_added_) and concentration (%). The two-stage anaerobic digestion was only reported in this study for co-digestion of both feedstocks for energy recovery from waste. This comparison showed that the two-stage system could improve the performance of methane production by enhancing the higher yield and concentration. In line with the previous reports (Chu et al., 2020; Yang et al., 2023), the two-stage AD could give the specific optimum conditions for both hydrogen-producing bacteria and methanogens in different containments due to favorable conditions for hydrolytic-acidogenic bacteria, methanogenesis may be suppressed, particularly at low pH levels. In certain cases, the methane concentration obtained exceeded the levels in this study when co-digesting NG with elephant (Rangseesuriyachai et al., 2023) and chicken manure (Yodthongdee et al., 2019). Nevertheless, the methane production yield still surpassed those of the two feedstocks. Recent studies involving food waste co-digestion with a variety of wastes exhibited a lower methane production yield than this study (Liu X. et al., 2022; Kosheleva et al., 2023; Lee et al., 2023; Yang et al., 2023). Alkaline pretreatment of food waste used for co-digestion with sewage sludge in two-stage anaerobic digestion (Lee et al., 2023) resulted in lower methane yield production. According to the comparison, the most suitable condition for methane production performance and effective energy recovery from these feedstocks is the combination of Napier grass and pretreated food waste with subcritical water hydrolysis in two-stage anaerobic digestion.

**TABLE 3:** Comparison of methane production performances with the previous studies on Napier grass and food waste.

No.	Substrate	Pretreatment	AD system	Temp. (°C)	CH_4_ yield (mL/g VS_added_)	CH_4_ conc. (%)	Reference
1	Napier grass + Food waste hydrolysate (1:1)	Untreated; Subcritical water hydrolysis	Batch	37	614.4	67.3	This study
			Two-Stage				
2	Napier grass + Food waste (1:4)	Untreated; Untreated	Batch	30	411	56.1	Boonpiyo et al. (2018)
			Single Stage				
3	Napier silage + Food waste (3:2)	Fermentation; Untreated	Batch	30	362	-	Boonpiyo et al. (2018)
			Single Stage				
4	Treated Napier grass + Food waste (1:3)	Alkaline pretreatment: Untreated	Batch	-	285	-	Pinpatthanapong et al. (2022)
			Single Stage				
5	Napier grass + Elephant dung (1:1)	Enzyme pretreatment; Untreated	Batch	35	234.8	-	Rangseesuriyachai et al. (2023)
			Single Stage				
6	Napier grass + Chicken manure (5.7:1)	Untreated; Untreated	Batch	46	492	73.9	Yodthongdee et al. (2019)
			Single Stage				
7	Mixed straw + Food waste (1:5)	Untreated; Untreated	Batch	35	580	67.6	Yong et al. (2015), Shrestha et al. (2023)
			Single Stage				
8	Synthetic food waste + Bioplastic (98:2)	Untreated; Untreated	Semi-continuous	37	331	57.3	Kosheleva et al. (2023)
			Single Stage				
9	Food waste + Sewage sludge (5 + 1)	Alkaline pretreatment	Batch	55	481.9	-	Lee et al. (2023)
		Alkaline pretreatment	Two-Stage				
10	Food waste + Chicken manure (17:3)	Untreated; Untreated	Batch	37	288.7	-	Liu et al. (2022b)
			Two-Stage				

### 3.2 SCOD, VS removal and VFA analysis for methane production

The SCOD and VS removal characteristics of the different substrate ratios are shown in Table 2. The SCOD removal efficiency of all digesters was in the range of 30.5%–68.5% in the co-digestion system and 46.0%–50.4% in the mono-digestion. The nutrients for bacterial cell growth, in the form of SCOD, enter the fermentation process as simple short-chain compounds after hydrolysis. The decrease in SCOD content in the mixtures indicates that the organic chemical has also been reduced. The removal of soluble chemical oxygen demand is a significant indicator of the effectiveness of anaerobic digestion in processing liquid substrates. Generally, stable anaerobic digestion achieves an SCOD removal rate of 80% or higher (Huang et al., 2017). Related to SCOD, the VS content represents the concentration of biodegradable organic matter in the mixtures, which can be assumed to be bacterial cells. Unfavorable environmental conditions can affect the inhibition of bacterial growth, resulting in the failure of the AD system (Hidalgo et al., 2015). The SCOD and VS removal data could be used as a basis for convincing the optimum NG:FW ratio in this study. As the maximum variation for methane production, the 1:1 ratio of NG:FW results in 45.1% of SCOD removal and only 5.7% VS removal. Compared to other variations, the SCOD removal performance was not the best. However, the lower VS removal provides promising support for condition C (1:1 ratio) to become the optimal ratio. As mentioned earlier, the low VS removal correlated with a favorable condition for bacterial growth. These results support the methane yield data where variation C (1:1) produced the highest among all co-digestion conditions (614.37 mL/g VS_added_ or 235 mL/g COD). The presence of untreated NG in the mixture could reduce the acidic inhibition effect in the second stage and create more favorable conditions for methane production. However, this would result in less hydrogen production in the first stage. However, the FW provides more sugar for hydrogen and organic acid in the mixture, resulting in a lower methane yield (refer to Table 2). It is necessary to explore the optimal ratio between NG and FW for two-stage anaerobic digestion due to the significant impact on hydrogen and methane yield. Although it has a lower bacterial cell degradation in the system, the conversion of nutrients into desired products, namely, hydrogen and methane, is also ineffective. A significant approach is required to improve the conversion of feedstock into energy. Prolonged fermentation incubation may be effective due to the use of untreated lignocellulosic biomass in the mixture which is probably most derived from the untreated Napier grass.

The content of VFAs in the mixture could represent the methane production performance in the AD system. As mentioned above, the acetoclastic methanogens require the presence of acetate, one of the organic acids grouped as VFAs, for methane biosynthesis. However, the high concentration of VFAs in the mixture could also cause system failure by acidifying the reactor, which must be avoided to maintain methanogen activity. The high-performance liquid chromatography (HPLC) can perform rapid and simultaneous qualitative and quantitative analysis of samples containing various solute components (Klimek-Turek et al., 2016), including for detecting the VFAs and sugar content. The sugar and VFAs profile for the initial and final second stage of methane production was shown in Table 4. The propionate and butyrate, which were produced during acidogenesis, were increased after fermentation in all conditions. In contrast, acetate, citrate, and glucose were significantly decreased after fermentation in all the conditions. These results showed that the VFAs, especially the acetate, will be highly correlated with methane production in the second stage of the AD system due to the activity of methanogens in producing the methane (Detman et al., 2021; Matambo et al., 2022).

**TABLE 4:** VFA analysis for co-digestion of methane production process.

Sample	Glucose		Citric acid		Acetic acid		Propionic acid		Butyric acid	
	Initial	Final	Initial	Final	Initial	Final	Initial	Final	Initial	Final
**A**	59.5 ± 0.0	0.0	9.0 ± 0.01	57.1 ± 0.1	1088.6 ± 2.4	0.0 ± 0.00	0.0 ± 0.0	1301.4 ± 2.6	3.9 ± 0.01	55.7 ± 0.2
**B**	39.2 ± 0.0	0.0	12.9 ± 0.02	53.5 ± 0.1	1117.8 ± 2.5	379 ± 0.83	0.0 ± 0.0	164.5 ± 0.3	147.9 ± 0.52	2496.7 ± 8.8
**C**	0.0 ± 0.0	0.0	118.5 ± 0.19	47.6 ± 0.1	587.5 ± 1.3	6.2 ± 0.01	81.1 ± 0.2	1295.6 ± 2.6	110.4 ± 0.39	4474.7 ± 15.7
**D**	0.0 ± 0.0	0.0	75.3 ± 0.12	31.0 ± 0.1	418.5 ± 0.9	0.0 ± 0.00	47.1 ± 0.1	731.4 ± 1.5	74.2 ± 0.26	2388.1 ± 8.4
**E**	0.0 ± 5.1	0.0	97.3 ± 0.16	51.1 ± 0.1	265.9 ± 0.6	2.6 ± 0.01	42.1 ± 0.1	1169 ± 2.4	71.1 ± 0.25	2363.2 ± 8.3
**NG**	0.0 ± 0.0	0.0	98.2 ± 0.16	78.1 ± 0.1	705.3 ± 1.5	6.3 ± 0.01	859.8 ± 1.7	1040.7 ± 2.1	0.0 ± 0.00	0.0 ± 0.0
**FW**	0.9 ± 0.0	0.0	15.6 ± 0.03	32.7 ± 0.1	750.5 ± 1.6	0.0 ± 0.00	0.0 ± 0.0	1096.2 ± 2.2	4.8 ± 0.02	1537.2 ± 5.4
**Control**	0.0 ± 0.0	0.0	64.1 ± 0.10	65.5 ± 0.1	94.0 ± 0.2	3.4 ± 0.01	0.0 ± 0.0	811.6 ± 1.6	296.5 ± 1.04	3843.2 ± 13.5

The increase of propionic acid at the end of fermentation is probably related to two main factors. First, compared to other VFAs, propionic acid production from sugar is easier during the acidogenic bacteria in the mixed culture (Uhlenhut et al., 2018). The acidogenic process remained active after 60 days of incubation due to the availability of complex substances, such as cellulose and hemicellulose, from untreated Napier grass. Second, during methanogenesis, propionic acid is more difficult to degrade compared to acetic acid (Li et al., 2017). The complex substances that remain in the mixture may have provided the necessary sustenance for the butyric acid-producing bacteria to acidify the anaerobic digester while also supplying hydrogen for hydrogenotrophic methanogens. Theoretically, the butyric acid pathway could result in a greater hydrogen yield than the propionic acid pathway. Also, it has been observed that there is a common occurrence of switching pathways from the propionic acid pathway to the butyric acid or ethanol pathway due to the influence of pH changes. Furthermore, these conditions are more likely to lead to a significant increase in the production of butyric acid during fermentation (Fang et al., 2017; Shi et al., 2019). As a longer carbon chain compared to acetic acid, the degradation of propionic acid and butyric acid during the methanogenesis becomes more challenging. The acetate at the final incubation was decreased due to the activity of acetoclastic methanogen that converts acetate into methane and carbon dioxide. On the other hand, the propionate and butyrate remained produced and needed thermodynamically favorable conditions (negative value of ΔG, Gibbs free energy) for their degradation (Wu D. et al., 2022). The increased concentration of propionic and butyric acid suggests that the untreated NG and FW hydrolysate mixture was insufficiently converted during the 60-day incubation period.

### 3.3 Kinetic for biomethane production by modified gompertz model

The kinetic study was conducted to investigate the optimum condition for co-digestion of NG and FW with different ratios. The modified Gompertz model was used to simulate the biomethane production parameters, including the potential total methane production (*P*), the maximum production rate (*R*_*m*_), and the length of the lag phase (*λ*). The higher number of *P* and *R*_*m*_ were desirable, while the better for the lag phase (*λ*) remained lower. The parameters simulated with the modified Gompertz model are shown in Table 5. The *R* ^*2*^ in all conditions showed a value higher than 0.99, which means that the predicted and experimental data fit well. In general, the co-digestion approach could reduce the lag phase of methane production compared to the mono-digestion of each Napier grass and food waste. A shorter lag phase was more desirable because it will reduce the incubation time and also reduce the operating cost in the AD system. The co-digestion also increases the potential total methane produced coupled with a higher maximum production rate. The kinetics study results are also useful for the optimum NG: FW ratio selection in this study. In the kinetic study, variation D (2:3) observed had the highest potential on methane production at 605.6 mL/g VS_added_ which is higher than variation C. However, the D condition had around 38% lower maximum production rate compared to C (1:1) and approximately 2 days longer in the lag phase. Based on these results, the 1:1 ratio of NG-to-FW remained the optimum condition for methane production in the NG-FW co-digestion system.

**TABLE 5:** The kinetics study results using Modified Gompertz Model on methane production data.

Sample code	NG:FW ratio in g VS_added_	*P* (mL/g VS_added_)	*R*_*m*_ (mL/g VS_added_.Day^-1^)	*λ (Lag phase)* *(Day)*	*R* ^*2*^
A	4:1	536.2 ± 9.0	20.88 ± 1.05	8.01 ± 0.63	0.9925
B	3:2	544.5 ± 7.2	24.42 ± 1.16	8.46 ± 0.53	0.9940
C	1:1	575.3 ± 9.4	26.92 ± 1.66	8.82 ± 0.66	0.9905
D	2:3	605.6 ± 15	16.78 ± 0.63	11.48 ± 0.59	0.9952
E	1:4	543.9 ± 9.3	23.68 ± 1.40	8.97 ± 0.67	0.9909
NG	5:0	557.8 ± 5.1	26.02 ± 0.88	9.82 ± 0.36	0.9973
FW	0:5	618.5 ± 8.1	32.03 ± 1.77	9.75 ± 0.55	0.9933
Control	-	54.4 ± 1.0	1.93 ± 0.08	13.07 ± 0.54	0.9955

## 4 Conclusion

The co-digestion of untreated Napier grass with hydrolyzed food waste enhanced the methane yield in two-stage anaerobic digestion. The optimal ratio of NG:FW was 1:1 in a gram of VS, with the value of cumulative biogas and methane production at 1,161.33 mL/g VS_added_ and 614.37 mL/g VS_added_, respectively. The methane concentration was higher than 65% on day 20 and remained in the range between 65%–80% until the end of fermentation. The SCOD and VS removal were 45.1% and 5.7%, respectively, with higher potential methane production and maximum production rate and also a shorter lag phase. This study showed that the two-stage anaerobic digestion and subcritical water hydrolysis (SCWH) could enhance the methane yield in the co-digestion of NG and FW. This finding could be implemented to enhance the effectiveness of energy recovery from biomass waste (NG and FW) via biological processes in anaerobic conditions. The room for improvement is also widely open for this research topic area, especially in pretreating the Napier grass as a potential source for bioenergy production from second-generation feedstock.

## Data Availability

The original contributions presented in the study are included in the article/Supplementary material, further inquiries can be directed to the corresponding author.

## References

[B1] Abbas Y. Yun S. Mehmood A. Shah F. A. Wang K. Eldin E. T. (2023). Co-digestion of cow manure and food waste for biogas enhancement and nutrients revival in bio-circular economy. Chemosphere 311, 137018. 10.1016/j.chemosphere.2022.137018 36374782

[B2] Akcakaya M. Tuncay S. Icgen B. (2022). Two-stage anaerobic digestion of ozonated sewage sludge predominantly took over by acetotrophic methanogens with increased biogas and methane production. Fuel 317, 123434. 10.1016/j.fuel.2022.123434

[B3] Al Ajlouni M. F. (2022). Solid wastes management, biogas and compost generated from organic waste at Al-akaider landfill in Jordan. Adv. Image Video Process 10. 10.14738/aivp.103.12362

[B4] Antukh T. Lee I. Joo S. Kim H. (2022). Hydrogenotrophs-based biological biogas upgrading technologies. Front. Bioeng. Biotechnol. 10, 833482. 10.3389/fbioe.2022.833482 35557857 PMC9085624

[B5] Arutyunov V. Savchenko V. Sedov I. Arutyunov A. Nikitin A. (2022). The fuel of our future: hydrogen or methane? Methane 1, 96–106. 10.3390/methane1020009

[B6] Azam W. Khan I. Ali S. A. (2023). Alternative energy and natural resources in determining environmental sustainability: a look at the role of government final consumption expenditures in France. Environ. Sci. Pollut. Res. 30, 1949–1965. 10.1007/s11356-022-22334-z PMC9362472 35925458

[B7] Balakrishnan D. Manmai N. Ponnambalam S. Unpaprom Y. Chaichompoo C. Ramaraj R. (2023). Optimized model of fermentable sugar production from Napier grass for biohydrogen generation via dark fermentation. Int. J. Hydrogen Energy 48, 21152–21160. 10.1016/j.ijhydene.2022.12.011

[B8] Boonpiyo S. Sittijunda S. Reungsang A. (2018). Co-digestion of napier grass with food waste and napier silage with food waste for methane production. Energies 11, 3200. 10.3390/en11113200

[B9] Chen T.-H. Shen M.-Y. Chen C.-Y. Chen Y.-W. Wang L.-H. Chu C.-Y. (2023). Biogas production from food waste hydrolysate using a subcritical water pretreated process and pulp wastewater seed sludge. Sustain. Energy Technol. Assessments 59, 103392. 10.1016/j.seta.2023.103392

[B10] Choudhary A. Kumar A. Kumar S. (2022). “Co-digestion of lignocellulosic wastes with food waste for sustainable biogas production,” in Microbial Biotechnology for renewable and sustainable energy, 77–97. 10.1007/978-981-16-3852-7_4

[B11] Chu C.-Y. Vo T.-P. Chen T.-H. (2020). A novel of biohythane gaseous fuel production from pineapple peel waste juice in two-stage of continuously stirred anaerobic bioreactors. Fuel 279, 118526. 10.1016/j.fuel.2020.118526

[B12] Chu C.-Y. Zheng J.-L. Chen T.-H. Bhuyar P. (2021). High performance of biohydrogen production in packed-filter bioreactor via optimizing packed-filter position. Int. J. Environ. Res. Public Health 18, 7462. 10.3390/ijerph18147462 34299912 PMC8304059

[B13] Cohn Z. Latty T. Abbas A. (2022). Understanding dietary carbohydrates in black soldier fly larvae treatment of organic waste in the circular economy. Waste Manag. 137, 9–19. 10.1016/j.wasman.2021.10.013 34700286

[B14] Detman A. Bucha M. Treu L. Chojnacka A. Pleśniak Ł. Salamon A. (2021). Evaluation of acidogenesis products’ effect on biogas production performed with metagenomics and isotopic approaches. Biotechnol. Biofuels 14, 125. 10.1186/s13068-021-01968-0 34051845 PMC8164749

[B15] Donkor K. O. Gottumukkala L. D. Lin R. Murphy J. D. (2022). A perspective on the combination of alkali pre-treatment with bioaugmentation to improve biogas production from lignocellulose biomass. Bioresour. Technol. 351, 126950. 10.1016/j.biortech.2022.126950 35257881

[B16] Dzulkarnain E. L. N. Audu J. O. Wan Dagang W. R. Z. Abdul-Wahab M. F. (2022). Microbiomes of biohydrogen production from dark fermentation of industrial wastes: current trends, advanced tools and future outlook. Bioresour. Bioprocess. 9, 16. 10.1186/s40643-022-00504-8 PMC10991117 38647867

[B17] Fang W. Ye J. Zhang P. Zhu X. Zhou S. (2017). Solid-state anaerobic fermentation of spent mushroom compost for volatile fatty acids production by pH regulation. Int. J. Hydrogen Energy 42, 18295–18300. 10.1016/j.ijhydene.2017.04.148

[B18] Gomes M. G. Paranhos A. G. de O. Camargos A. B. Baêta B. E. L. Baffi M. A. Gurgel L. V. A. (2022). Pretreatment of sugarcane bagasse with dilute citric acid and enzymatic hydrolysis: use of black liquor and solid fraction for biogas production. Renew. Energy 191, 428–438. 10.1016/j.renene.2022.04.057

[B19] Govarthanan M. Manikandan S. Subbaiya R. Krishnan R. Y. Srinivasan S. Karmegam N. (2022). Emerging trends and nanotechnology advances for sustainable biogas production from lignocellulosic waste biomass: a critical review. Fuel 312, 122928. 10.1016/j.fuel.2021.122928

[B20] Gundupalli M. P. Cheenkachorn K. Chuetor S. Kirdponpattara S. Gundupalli S. P. Show P.-L. (2023). Assessment of pure, mixed and diluted deep eutectic solvents on Napier grass (Cenchrus purpureus): compositional and characterization studies of cellulose, hemicellulose and lignin. Carbohydr. Polym. 306, 120599. 10.1016/j.carbpol.2023.120599 36746569

[B21] He C. Song H. Liu L. Li P. Kumar Awasthi M. Xu G. (2022a). Enhancement of methane production by anaerobic digestion of corn straw with hydrogen-nanobubble water. Bioresour. Technol. 344, 126220. 10.1016/j.biortech.2021.126220 34715343

[B22] He X. Xu W. Lu J. Wu J. Guo Z. Wei X. (2022b). Enhanced direct interspecies electron transfer and methane production during anaerobic digestion of fat, oil, and grease by coupling carbon-based conductive materials and exogenous hydrogen. Bioresour. Technol. 364, 128083. 10.1016/j.biortech.2022.128083 36216280

[B23] Hidalgo D. Gómez M. Martín-Marroquín J. M. Aguado A. Sastre E. (2015). Two-phase anaerobic co-digestion of used vegetable oils’ wastes and pig manure. Int. J. Environ. Sci. Technol. 12, 1727–1736. 10.1007/s13762-014-0560-9

[B24] Huang C. Zhao C. Guo H.-J. Wang C. Luo M.-T. Xiong L. (2017). Fast startup of semi-pilot-scale anaerobic digestion of food waste acid hydrolysate for biogas production. J. Agric. Food Chem. 65, 11237–11242. 10.1021/acs.jafc.7b04005 29200277

[B25] Huang W. T. Zheng Z. C. Hua D. Chen X. F. Zhang J. Chen H. H. (2022). Adaptive responses of carbon and nitrogen metabolisms to nitrogen - deficiency in Citrus sinensis seedlings. BMC Plant Biol. 1, 370. 10.1186/s12870-022-03759-7 PMC9316421 35879653

[B26] Jiang M. Song S. Liu H. Dai X. Wang P. (2022). Responses of methane production, microbial community and antibiotic resistance genes to the mixing ratio of gentamicin mycelial residues and wheat straw in anaerobic co-digestion process. Sci. Total Environ. 806, 150488. 10.1016/j.scitotenv.2021.150488 34607101

[B27] Kaushal R. Sandhu S. Kumar Soni M. (2022). Anaerobic co-digestion of food waste, algae, and cow dung for biogas yield enhancement as a prospective approach for environmental sustainability. Sustain. Energy Technol. Assessments 52, 102236. 10.1016/j.seta.2022.102236

[B28] Klimek-Turek A. Sikora M. Rybicki M. Dzido T. H. (2016). Frontally eluted components procedure with thin layer chromatography as a mode of sample preparation for high performance liquid chromatography quantitation of acetaminophen in biological matrix. J. Chromatogr. A 1436, 19–27. 10.1016/j.chroma.2016.01.053 26839178

[B29] Kosheleva A. Gadaleta G. De Gisi S. Heerenklage J. Picuno C. Notarnicola M. (2023). Co-digestion of food waste and cellulose-based bioplastic: from batch to semi-continuous scale investigation. Waste Manag. 156, 272–281. 10.1016/j.wasman.2022.11.031 36521212

[B30] Kriswantoro J. A. Chu C.-Y. Chang T.-R. Pai H.-J. Chang C.-K. Chiu Y.-P. (2023). Comparison of thermal alkaline pretreatment and zinc acetate-catalyzed methanolysis (MtOH-ZnOAc) for anaerobic digestion of bioplastic waste. Bioresour. Technol. 377, 128959. 10.1016/j.biortech.2023.128959 36965583

[B31] Lee W. Mo K. Park C. Kim D. Park S. Lee D. (2023). Co‐digestion of food waste and sewage sludge using the combination of a thermal alkali pre‐treatment and a two‐stage anaerobic digestion system. J. Chem. Technol. Biotechnol. 98, 591–601. 10.1002/jctb.7133

[B32] Li R. Chen S. Li X. (2010). Biogas production from anaerobic Co-digestion of food waste with dairy manure in a two-phase digestion system. Appl. Biochem. Biotechnol. 160, 643–654. 10.1007/s12010-009-8533-z 19214795

[B33] Li W. Cheng C. Cao G. Yang S.-T. Ren N. (2019). Potential of hydrogen production from sugarcane juice by Ethanoligenens harbinense Yuan-3. J. Clean. Prod. 237, 117552. 10.1016/j.jclepro.2019.07.027

[B34] Li Y. Zhang Y. Kong X. Li L. Yuan Z. Dong R. (2017). Effects of ammonia on propionate degradation and microbial community in digesters using propionate as a sole carbon source. J. Chem. Technol. Biotechnol. 92, 2538–2545. 10.1002/jctb.5260

[B35] Litti Y. V. Potekhina M. A. Zhuravleva E. A. Vishnyakova A. V. Gruzdev D. S. Kovalev A. A. (2022). Dark fermentative hydrogen production from simple sugars and various wastewaters by a newly isolated Thermoanaerobacterium thermosaccharolyticum SP-H2. Int. J. Hydrogen Energy 47, 24310–24327. 10.1016/j.ijhydene.2022.05.235

[B36] Liu H. Wang X. Fang Y. Lai W. Xu S. Lichtfouse E. (2022a). Enhancing thermophilic anaerobic co-digestion of sewage sludge and food waste with biogas residue biochar. Renew. Energy 188, 465–475. 10.1016/j.renene.2022.02.044

[B37] Liu X. Yang Y. Wu N. Wei Y. Shan H. Zhao H. (2022b). Co-Production of biohydrogen and biomethane from chicken manure and food waste in a two-stage anaerobic fermentation process. Appl. Biochem. Biotechnol. 194, 3706–3720. 10.1007/s12010-022-03945-1 35499692

[B38] López-Aguilar H. A. Morales-Durán B. Quiroz-Cardoza D. Pérez-Hernández A. (2023). Lag phase in the anaerobic Co-digestion of Sargassum spp. and organic domestic waste. Energies 16, 5462. 10.3390/en16145462

[B39] Manokhoon P. Rangseesuriyachai T. (2020). Effect of two-stage sodium hydroxide pretreatment on the composition and structure of Napier grass (Pakchong 1) (Pennisetum purpureum). Int. J. Green Energy 17, 864–871. 10.1080/15435075.2020.1809425

[B40] Matambo T. Mutungwazi A. Awosusi A. (2022). Comparative functional microbiome profiling of various animal manures during their anaerobic digestion in biogas production processes. SSRN Electron. J. 10.2139/ssrn.4217101

[B41] Michelz Beitel S. Fontes Coelho L. Sass D. C. Contiero J. (2017). Environmentally friendly production of D(−) lactic acid by Sporolactobacillus nakayamae: investigation of fermentation parameters and fed-batch strategies. Int. J. Microbiol. 2017, 1–11. 10.1155/2017/4851612 PMC5610840 29081803

[B42] Milán Z. Montalvo S. Ilangovan K. Monroy O. Chamy R. Weiland P. (2010). The impact of ammonia nitrogen concentration and zeolite addition on the specific methanogenic activity of granular and flocculent anaerobic sludges. J. Environ. Sci. Heal. Part A 45, 883–889. 10.1080/10934521003709099 20419585

[B43] Nikitina A. A. Kallistova A. Y. Grouzdev D. S. Kolganova T. V. Kovalev A. A. Kovalev D. A. (2022). Syntrophic butyrate-oxidizing consortium mitigates acetate inhibition through a shift from acetoclastic to hydrogenotrophic methanogenesis and alleviates VFA stress in thermophilic anaerobic digestion. Appl. Sci. 13, 173. 10.3390/app13010173

[B44] Norouzi O. Dutta A. (2022). The current status and future potential of biogas production from Canada’s organic fraction municipal solid waste. Energies 15, 475. 10.3390/en15020475

[B45] Onyeaka H. Mansa R. F. Wong C. M. V. L. Miri T. (2022). Bioconversion of starch base food waste into bioethanol. Sustainability 14, 11401. 10.3390/su141811401

[B46] Pinpatthanapong K. Boonnorat J. Glanpracha N. Rangseesuriyachai T. (2022). Biogas production by co-digestion of sodium hydroxide pretreated Napier grass and food waste for community sustainability. Energy Sources, Part A recover. Util. Environ. Eff. 44, 1678–1692. 10.1080/15567036.2022.2055232

[B47] Pomdaeng P. Chu C.-Y. Sripraphaa K. Sintuya H. (2022). An accelerated approach of biogas production through a two-stage BioH2/CH4 continuous anaerobic digestion system from Napier grass. Bioresour. Technol. 361, 127709. 10.1016/j.biortech.2022.127709 35905883

[B48] Pomdaeng P. Kongthong O. Tseng C.-H. Dokmaingam P. Chu C.-Y. (2023). An immobilized mixed microflora approach to enhancing hydrogen and methane productions from high-strength organic loading food waste hydrolysate in series batch reactors. Int. J. Hydrogen Energy. 52, 160–169. 10.1016/j.ijhydene.2023.09.187

[B49] Qian L. Rao Q. Liu H. McCarthy B. Liu L. X. Wang L. (2022). Food waste and associated carbon footprint: evidence from Chinese universities. Ecosyst. Heal. Sustain. 8. 10.1080/20964129.2022.2130094

[B50] Rahman A. Shahazi R. Noureen S. Nova B. Uddin M. R. Hossain S. (2021). Biogas production from anaerobic co - digestion using kitchen waste and poultry manure as substrate — part 1: substrate ratio and effect of temperature. Biomass Convers. Biorefinery 13, 6635–6645. 10.1007/s13399-021-01604-9 PMC8189274 34127942

[B51] Rakau M. V. Fushai F. Baloyi J. J. (2022). Nutritive value of mixed napier grass (Pennisetum purpureum)- Leucaena (Leucaena leucocephala) silage for ruminants. Anim. Nutr. Feed Technol. 22, 433–448. 10.5958/0974-181X.2022.00034.8

[B52] Rangseesuriyachai T. Boonnorat J. Glanpracha N. Khetkorn W. Thiamngoen P. Pinpatthanapong K. (2023). Anaerobic co-digestion of elephant dung and biological pretreated Napier grass: synergistic effect and kinetics of methane production. Biomass Bioenergy 175, 106849. 10.1016/j.biombioe.2023.106849

[B53] Saidi R. Hamdi M. Bouallagui H. (2023). Enhanced hydrogen and methane production from date fruit wastes using semi continuous two-stage anaerobic digestion process with increasing organic loading rates. Process Saf. Environ. Prot. 174, 267–275. 10.1016/j.psep.2023.04.018

[B54] Sawasdee A. Haosagul S. Pisutpaisal N. (2023). Biogas production from Co-digestion between rice straw and food waste with pilot scale. Int. J. Environ. Res. 17, 31. 10.1007/s41742-023-00523-z

[B55] Shi E. Li J. Zhang M. (2019). Application of IWA Anaerobic Digestion Model No. 1 to simulate butyric acid, propionic acid, mixed acid, and ethanol type fermentative systems using a variable acidogenic stoichiometric approach. Water Res. 161, 242–250. 10.1016/j.watres.2019.05.094 31202111

[B56] Shrestha S. Pandey R. Aryal N. Lohani S. P. (2023). Recent advances in co-digestion conjugates for anaerobic digestion of food waste. J. Environ. Manage. 345, 118785. 10.1016/j.jenvman.2023.118785 37611516

[B57] Simioni T. Agustini C. B. Dettmer A. Gutterres M. (2022). Enhancement of biogas production by anaerobic co-digestion of leather waste with raw and pretreated wheat straw. Energy 253, 124051. 10.1016/j.energy.2022.124051

[B58] Sohrab Hossain M. Mokarram Badsha M. Balakrishnan V. Shaharun M. S. (2023). A review on bio-hydrogen production from food waste: potential and challenges. in Proceedings of the 1st international conference of new energy, 105–114. 10.1007/978-981-99-0859-2_12

[B59] Soltaninejad A. Jazini M. Karimi K. (2022). Biorefinery for efficient xanthan gum, ethanol, and biogas production from potato crop residues. Biomass Bioenergy 158, 106354. 10.1016/j.biombioe.2022.106354

[B60] Song Y. Pei L. Chen G. Mu L. Yan B. Li H. (2023). Recent advancements in strategies to improve anaerobic digestion of perennial energy grasses for enhanced methane production. Sci. Total Environ. 861, 160552. 10.1016/j.scitotenv.2022.160552 36511320

[B61] Thaemngoen A. Saritpongteeraka K. Leu S.-Y. Phuttaro C. Sawatdeenarunat C. Chaiprapat S. (2020). Anaerobic digestion of napier grass (pennisetum purpureum) in two-phase dry digestion system versus wet digestion system. BioEnergy Res. 13, 853–865. 10.1007/s12155-020-10110-1

[B62] Thongbunrod N. Chaiprasert P. (2023). Anaerobic microbial cocktail of lignocellulolytic fungi and bacteria with methanogens for boosting methane production from unpretreated rice straw. Bioprocess Biosyst. Eng. 46, 251–264. 10.1007/s00449-022-02829-2 36495340

[B63] Uhlenhut F. Schlüter K. Gallert C. (2018). Wet biowaste digestion: ADM1 model improvement by implementation of known genera and activity of propionate oxidizing bacteria. Water Res. 129, 384–393. 10.1016/j.watres.2017.11.012 29174828

[B64] Wu D. Li L. Zhen F. Liu H. Xiao F. Sun Y. (2022a). Thermodynamics of volatile fatty acid degradation during anaerobic digestion under organic overload stress: the potential to better identify process stability. Water Res. 214, 118187. 10.1016/j.watres.2022.118187 35184016

[B65] Wu Q. Zou D. Zheng X. Liu F. Li L. Xiao Z. (2022b). Effects of antibiotics on anaerobic digestion of sewage sludge: performance of anaerobic digestion and structure of the microbial community. Sci. Total Environ. 845, 157384. 10.1016/j.scitotenv.2022.157384 35843318

[B66] Xiao Y. Zan F. Zhang W. Hao T. (2022). Alleviating nutrient imbalance of low carbon-to-nitrogen ratio food waste in anaerobic digestion by controlling the inoculum-to-substrate ratio. Bioresour. Technol. 346, 126342. 10.1016/j.biortech.2021.126342 34785330

[B67] Xu F. Okopi S. I. Jiang Y. Chen Z. Meng L. Li Y. (2022). Multi-criteria assessment of food waste and waste paper anaerobic co-digestion: effects of inoculation ratio, total solids content, and feedstock composition. Renew. Energy 194, 40–50. 10.1016/j.renene.2022.05.078

[B68] Xu Q. Liao Y. Cho E. Ko J. H. (2020). Effects of biochar addition on the anaerobic digestion of carbohydrate-rich, protein-rich, and lipid-rich substrates. J. Air Waste Manage. Assoc. 70, 455–467. 10.1080/10962247.2020.1733133 32091971

[B69] Yadav M. Balan V. Varjani S. Tyagi V. K. Chaudhary G. Pareek N. (2023). Multidisciplinary pretreatment approaches to improve the bio-methane production from lignocellulosic biomass. BioEnergy Res. 16, 228–247. 10.1007/s12155-022-10489-z

[B70] Yang S. Luo F. Yan J. Zhang T. Xian Z. Huang W. (2023). Biogas production of food waste with *in-situ* sulfide control under high organic loading in two-stage anaerobic digestion process: strategy and response of microbial community. Bioresour. Technol. 373, 128712. 10.1016/j.biortech.2023.128712 36758645

[B71] Yodthongdee S. Weerayutsil P. Khuanmar K. (2019). Application of combined mixture process design for enhancement of methane production using Co-digestion of chicken manure and napier grass. J.Mech.Cont.and Math. Sci., 85–96. 10.26782/jmcms.2019.03.00008

[B72] Yong Z. Dong Y. Zhang X. Tan T. (2015). Anaerobic co-digestion of food waste and straw for biogas production. Renew. Energy 78, 527–530. 10.1016/j.renene.2015.01.033

[B73] Yun Y.-M. Lee M.-K. Im S.-W. Marone A. Trably E. Shin S.-R. (2018). Biohydrogen production from food waste: current status, limitations, and future perspectives. Bioresour. Technol. 248, 79–87. 10.1016/j.biortech.2017.06.107 28684176

